# Prolactin and heat stress; focus on domestic ruminants

**DOI:** 10.1093/jas/skaf020

**Published:** 2025-02-05

**Authors:** Iain J Clarke, Frank R Dunshea, Surinder S Chauhan

**Affiliations:** School of Agriculture, Food and Ecosystem Sciences, Faculty of Science, The University of Melbourne, Royal Parade, Parkville, Melbourne, VIC 3010, Australia; School of Agriculture, Food and Ecosystem Sciences, Faculty of Science, The University of Melbourne, Royal Parade, Parkville, Melbourne, VIC 3010, Australia; School of Biology, Faculty of Biological Sciences, The University of Leeds, Leeds LS2 9JT, UK; School of Agriculture, Food and Ecosystem Sciences, Faculty of Science, The University of Melbourne, Royal Parade, Parkville, Melbourne, VIC 3010, Australia

**Keywords:** heat stress, prolactin, ruminants

## Abstract

Prolactin has traditionally been associated with milk production, but recent studies identify prolactin as having many other functions. These include a role in pelage growth, sweating, immune function, metabolism, and water/electrolyte balance. A signature of heat stress HS is a rise in prolactin concentrations so the question arises as to whether this hormone has a particular function in relation to response to or mitigation of HS. Thus, prolactin plays a multifaceted role in the physiological and behavioral responses of livestock to HS, contributing to their ability to cope with warmer temperatures and maintain homeostasis. A major advance in recent years is the identification of the *SLICK* gene in cattle, being a mutation in the prolactin receptor. It is responsible for a phenotype of short, shiny coat. *SLICK* confers heat resilience and offers a realistic means of mitigating HS by introgression into cattle without the mutation. The purpose of this article is to ascertain what functions prolactin may have in the response to HS. It appears that prolactin may be involved in many of the physiological processes that are affected by HS, but it is clear that definitive evidence of cause/effect is yet to be discerned.

## Graphical Abstract

**Graphical Abstract:**
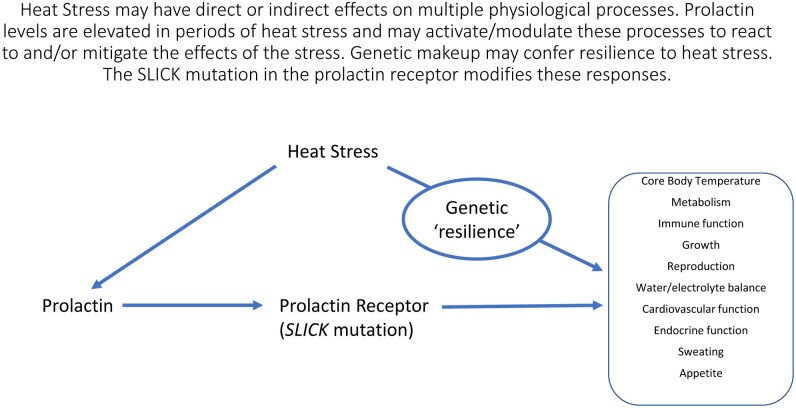

## Teaser

We review the role of prolactin in the response to heat stress, across a range of bodily processes. In addition, we highlight the role of prolactin in heat stress by reviewing the impact of the SLICK mutation to confer resilience to heat stress.

## Introduction

Prolactin (PRL) is a somewhat enigmatic hormone, originally identified as a protein that was lactogenic in pigeons (Riddle et al., 1932). For many years, the role of PRL in the development of the mammary glands and lactogenesis was studied without particular reference to its broader functions (Falconer, 1980). It is now apparent that PRL is produced by many organs in the body (Freeman et al., 2000; Marano and Ben-Jonathan, 2014) and serves as many as 300 functions (Bernard et al., 2019) including a range of homeostatic and homeorhetic functions. In addition to the direct effects of PRL, it was first proposed (Bauman and Currie, 1980) that PRL was one of a few candidate homeostatic hormones that might exert some of their effects through changing tissue responsiveness to other signals. The following review focusses on the role of PRL in the response to heat stress (HS), especially in domestic ruminants. To fully understand this particular function, it is necessary to consider factors regulating PRL secretion to indicate its wider roles in the regulation of the immune system, metabolic function, and sweating as well as taking into consideration the diurnal and circannual rhythms of PRL secretion.

## Prolactin as a Hormone

Prolactin (PRL) is a single-chain polypeptide protein hormone, encoded by a single gene and secreted in a pulsatile manner by the lactotropes of the anterior pituitary gland (McNeilly and Chard, 1974). Prolactin and shorter PRL variants are expressed in a range of non-pituitary tissues and the extent to which PRL exerts effect depends on these variants and the means of control over expression of the same (Foitzik et al., 2009). As pointed out by these authors, the pituitary-specific transcription factor, *PIT-1*, is essential for PRL production in anterior pituitary lactotropes, but synthesis in extrapituitary tissues is independent of *PIT-1*. Thus, stimulation of production that may occur under HS will rely on *PIT-1* in the pituitary and alternative transcription factors in extrapituitary tissues (reviewed in (Foitzik et al., 2009).

There are many excellent reviews on the production of PRL and its control and the reader is referred to two in particular (Freeman et al., 2000; Goffin et al., 2002)

## Control of Prolactin Secretion

In sheep, as in other seasonal species, PRL secretion is dependent upon photoperiod, such that it is greater during long days than during short days (Ravault, 1976; Kennaway et al., 1983). Photoperiodism is driven by the pattern of melatonin secretion, which is diurnal; being greater during hours of darkness (Kennaway et al., 1983). The control of the secretion of PRL is somewhat different to that of other anterior pituitary hormones in that there is no known specific releasing factor for the hormone, as there is for gonadotropins, GH, thyroid stimulating hormone, and adrenocorticotropin. In fact, whereas the secretion of hormones such as gonadotropins is eliminated by disconnection of the pituitary gland from the hypothalamus (hypothalamo-pituitary disconnection—HPD), PRL concentrations are actually greater following the procedure, indicating that it is under tonic inhibitory tone (Thomas et al., 1986). The surgical procedure of HPD involves extirpation of the tissue above the median eminence (ME) and the placement of a barrier between the ME and the hypothalamus. Preserving the ME and the anterior pituitary as a unit enables the preservation of vital blood supply to the latter (Clarke et al., 1983).

Interestingly, the influence of photoperiod on the secretion of PRL is preserved following HPD, leading to the notion that factors arising from the pars tuberalis of the ME are involved in the photoperiodic effect (Lincoln and Clarke, 1994). The pars tuberalis, which is the outer zone of the ME, contains cells that express melatonin receptors (Morgan et al., 1989), so the hypothesis seems reasonable. Since the photoperiodic effect on PRL secretion is dependent upon the diurnal pattern of melatonin secretion, elimination of the melatonin signal (by superior cervical ganglionectomy) prevented the change in secretion following the transition from short-day to long-day photoperiod in Soay rams (Lincoln et al., 2006).

Despite there being evidence that various factors stimulate PRL secretion, these are not specific (Thomas et al., 1987, 1988a). For example, thyrotropin-releasing hormone (TRH) is a potent releasor of PRL (Thomas et al., 1988b), albeit the specific releasing factor for thyrotropin. Although TRH can evoke a brisk elevation in PRL secretion, the pulsatile secretion of this hypothalamic peptide into the hypophysial portal blood of the sheep does not correlate with pulsatile secretion of PRL (Thomas et al., 1988b). It seems most likely that positive drive for PRL secretion is due to several factors although it is intriguing to note that following HPD, pulsatile secretion of the hormone persists.

A large body of evidence indicates that the secretion of dopamine from the hypothalamus exerts inhibitory control on the lactotropes (Freeman et al., 2000), although measurement of dopamine in the long hypophysial portal blood of sheep did not provide evidence for this (Thomas et al., 1989b). Alternatively, it is possible that dopamine could reach the anterior pituitary gland via the short portal vessels and the posterior lobe of the pituitary. Certainly, there is overwhelming evidence that agonists and antagonists of the D2 dopamine receptor respectively suppress or enhance the secretion of PRL in sheep (Thomas et al., 1989a) as in other species but this could be due, at least in part, to an effect at the concentration of the hypothalamus rather than the pituitary gland (Curlewis et al., 1993).

## Influence of Photoperiod and Environmental Temperature on PRL Concentrations

**Figure 1.**
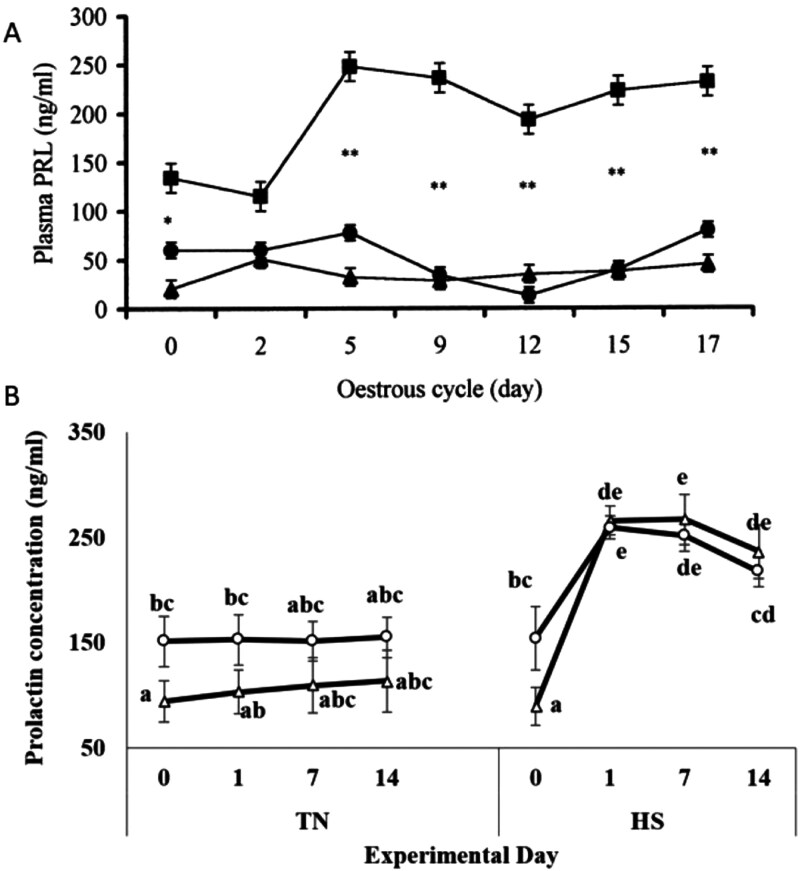
Panel A: Mean (± SEM) plasma PRL concentrations in heifers kept in thermal comfort (TC ●) (18 °C), thermal comfort with restricted feeding (TCRF) or increased ambient temperature (HAT) (32 °C) in climate chambers and measured across the estrous cycle. *P < 0.05 and **P < 0.01 between HAT and TC. Adapted from Ronchi et al. 2001 (Ronchi et al., 2001), with permission. Panel B: Mean (± SEM) PRL concentrations in Dorper (△) and Crossbred-Merino (O) lambs in climate chambers and subjected to either thermoneutral (TN; 18-21 °C) or cyclic HS (HS; 28°- 40 °C Values with different letters differ significantly (breed; P < 0.05, treatment; P < 0.001; day; P < 0.001). Reproduced from Joy et al. (2020), with permission.

As indicated above, PRL affects many bodily functions (Freeman et al., 2000), such as regulating the immune system. The focus of our review, however, is the role that PRL plays in HS in ungulates with special consideration on the effect of HS and the consequential rise in PRL concentrations. As indicated above, PRL concentrations in blood are strongly influenced by photoperiod, being greater during long days. Because summer is the time of long days as well as warmer ambient temperatures, it is difficult to separate these two factors (photoperiod and temperature) as being causative of the increased PRL concentrations. One way to separate these two factors would be to determine whether elevated temperatures per se affect the function of the pituitary lactotropes in animals during long days in controlled experiments. A rise in circulating PRL concentrations is a signature of HS in a wide range of species, including ungulates (Alamer, 2011) (Figure 1). As such, it includes an increase in the synthesis and secretion of PRL, including the PRL response to TRH. As to whether there is a combined effect of temperature and photoperiod, a particular experimental approach is required, testing the effect of variation in temperatures in long- and short-day photoperiod. It is not a trivial consideration as to whether HS increases PRL secretion equally in both long day and short-day photoperiod because in the advent of climate change, warmer ambient temperatures may be experienced during winter months (short days). Whereas most studies on grazing species indicate the time of year the experimental work was done, this does not allow any determination of whether the effect of or the response to HS differs under differing external photoperiods. This is notwithstanding the fact that experiments conducted on animals taken at a particular time of year and performed under artificial conditions (e.g., in climate chambers) can control photoperiod and temperature in the short term. Carryover effects of the seasonal photoperiod may prevail over the short-term photoperiod imposed in climate chambers. Time of day is another consideration, since PRL concentrations are increased at night (Karasek et al., 1990). The nighttime rise in PRL may be linked to the nocturnal rise in melatonin but as to whether this is cause or effect remains unclear (Karasek et al., 2004). Exercise also evokes a rise in PRL concentrations, due to hyperthermia (Radomski et al., 1998). Both need to be taken into consideration, when evaluating the role of PRL in HS.

## Prolactin Receptor

Prolactin acts through a single membrane-bound receptor that belongs to the Class 1 cytokine receptor superfamily and its characteristics are detailed in extensive reviews (Bole-Feysot et al., 1998; Freeman et al., 2000; Harris et al., 2004). Cloning of the rat PRL receptor was originally published in 1998 (Boutin et al., 1988) and it was soon found to exist in multiple isoforms, encoded by a single gene displaying multiple promoter usage and 3’-exon splicing el. (Kelly et al., 1989; Ormandy et al., 1998). Whereas there are various isoforms of the receptor, the extracellular domain is conserved and the isoforms vary in the length and composition of the cytoplasmic domains, leading to what are referred to as short and long isoforms (Kelly et al., 1991, 1993; Bole-Feysot et al., 1998). Binding of PRL to the long form of the receptor activates STAT5 through membrane-associated JAK2, but the short form of the receptor may signal through other pathways, such as MAPK (Harris et al., 2004).

The PRL receptor is widely distributed throughout various tissues in the body, being highly expressed in the mammary gland, ovary, and the brain. In addition, the receptor is expressed in the pituitary gland, heart, lung, thymus, spleen, liver, pancreas, kidney, adrenal gland, uterus, skeletal muscle, and skin (Nagano and Kelly, 1994; Bole-Feysot et al., 1998; Foitzik et al., 2009).

## Prolactin, Hair/Wool Growth and Shedding

Prolactin transcripts and protein are found in the hair follicle, in mice (Craven et al., 2001) and humans (Foitzik et al., 2003, 2006). Of particular interest is the observation that PRL binding was localized to the dermal papillae of wool follicles and in sebaceous and apocrine sweat glands in ovine skin (Choy et al., 1995; Nixon et al., 2002) discussed in detail below.

Wool growth in domesticated sheep breeds that do not shed their wool is consistent throughout the year, but in shedding breeds which are largely considered to be ‘primitive’ and not subjected to artificial selection pressure show a distinct annual cycle of wool growth, shedding their wool on an annual basis (Lincoln, 1990). So-called “wild” or “ancient” breeds, such as Soay and Mouflon are relatively unselected and display the shedding property. One might consider the Wiltshire and Dorper breeds, which display annual shedding, to be ‘domesticated’ being selected for performance traits. The name of the former breed is somewhat misleading because the origin is in the in the Mediterranean or Middle East being brought to England by the Romans. Dorper is a composite breed developed in South Africa being a cross between Dorset Horn and Persian Blackface breeds, the latter conferring the shedding property. In shedding breeds, such as Mouflon and Wiltshire Horn, the molt is linked to the circannual change in PRL concentrations (Lincoln, 1990; Pearson et al., 1996). Other composite breeds such as the Easycare (Welsh Mountain × Wiltshire Horn) and Aussie White (White Dorper × Van Rooy × Poll Dorset × Texel) display shedding, due to their inheritance of a dominant autosomal gene (Pollott, 2011). A GWAS study of Easy-care sheep identified 4 SNP that may be associated with shedding (Matika et al., 2013) but the authors were cautious to point out that the SNP may not fully explain the genetics of shedding. Cyclic changes in photoperiod altered plasma PRL concentrations, wool follicle activity, and PRL receptor expression in a related fashion, suggesting that PRL functions to alter seasonal patterns of wool growth (Nixon et al., 2002). Other studies in goats yielded dichotomous results. Thus, the culture of hair follicles from Cashmere goats showed that PRL stimulated the elongation of the hair shafts. In two studies (Ibraheem et al., 1994; Puchala et al., 2003) infused PRL into the superﬁcial branches of the deep circumﬂex iliac artery and vein of Angora goats with saline infusion as control, showing that the PRL reduced mohair fiber growth. The latter study is consistent with the weight of evidence that seasonal increase in PRL concentrations reduces hair/wool growth but more work is required to understand the exact means by which PRL exerts such an effect. As a general rule, the weight of evidence is that molting in shedding breeds occurs when PRL concentrations rise with increasing seasonal daylength (Lincoln, 1990; Pearson et al., 1996).

In cattle, the relationship between hair growth and PRL was somewhat elucidated by a seminal study by Littlejohn et al (Littlejohn et al., 2014). Thus, a mutation in the PRL gene per se produces a hairy phenotype in cattle, whereas a mutation in the PRL receptor produces the *SLICK* phenotype discussed below. If it is accepted that PRL inhibits hair/wool growth, how is it possible that a mutation in the receptor leads to the *SLICK* phenotype? One answer may be that the mutation confers a gain-of-function, enhancing the effect of PRL to reduce hair growth.

## Sweating

The sweat glands can be characterized to be of two types, being eccrine and apocrine and a detailed description of the anatomy of the two types is provided by Bligh (Bligh, 1967). Whereas eccrine glands produce watery sweat from non-hairy regions humans and some other species, domesticated animals, including horses, cows, sheep, and goats, in the human and some domesticated species including horses, cows, sheep, and goats produce sweat from apocrine glands. Eccrine glands develop during the fetal life of humans, whereas apocrine glands develop at the time of puberty. In humans, eccrine glands open to the skin surface and are numerous. Apocrine glands open into hair sheath, are localized to specific regions, and produce a protein/lipid-rich sweat. Eccrine glands produce a watery sweat and are most involved in thermoregulation by evaporative cooling in humans (Baker, 2019).

Eccrine glands are under cholinergic control, whereas apocrine glands are under the control of circulating adrenalin, which is increased with exercise and under HS (Evans, 1966). Patterns of sweating in sheep, goats, and cattle are summarized by Bligh (Bligh, 1967). Horses produce copious amounts of sweat in response to elevated ambient temperature or exercise, but the same is not true for the other above-mentioned species. Detailed studies by Evans (Evans, 1966) and others cited by the same, showed that the apocrine glands of the horse were not innervated by adrenergic nerves but respond to adrenalin, leading to the conclusion that under conditions such as exercise, a rise in circulating adrenalin stimulates sweating. Interestingly, pigs possess apocrine glands that respond to adrenergic stimulation, but the openings of the glands are generally blocked by keratin and sweating is not observed, except in the snout, in which eccrine glands are found (Ingram, 1967).

**Figure 2.**
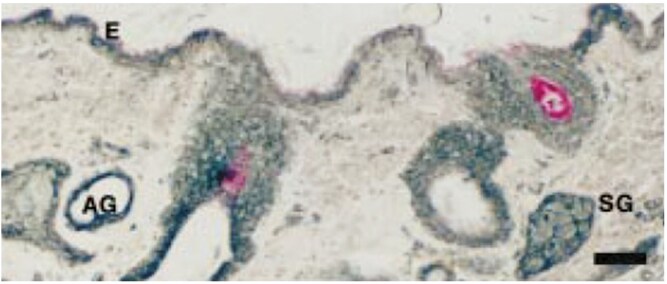
Staining of prolactin receptor in apocrine sweat gland (AG) and sebaceous gland (SG) in interfollicular epidermis (E) of ovine skin using silver-enhanced immunogold immunohistochemistry. Dark staining indicates the presence of the receptor. Bar represents 50 μm. Taken from Choy et al (1997), with permission.

The extent to which PRL acts to regulate sweating in domestic animals is an issue of ongoing investigation. Prolactin or PRL-like immunoreactivity was localized to sweat glands in human skin, being localized in clear cells that are considered to regulate fluid secretion (Walker et al., 1989). Elucidation of such a display was prompted by the earlier demonstration that PRL regulates osmoregulation in teleost fish (Loretz and Bern, 1982). In humans, it was shown that PRL can change the chloride ion concentration in sweat (Robertson et al., 1986), but such a mechanism is not relevant to the domestic species in which eccrine glands do not predominate. As mentioned above, PRL binding was localized to the dermal papillae of wool follicles and in sebaceous and apocrine sweat glands in ovine skin (Choy et al., 1995) (Figure 2). Until it can be demonstrated that PRL regulates sweating from apocrine glands in domesticated species, it is impossible to ascribe a function of the hormone. On the other hand, it would be interesting to ascertain whether PRL regulates the copious exercise-induced sweating demonstrated by horses and in cattle, which also display significant sweat response to warm/hot ambient temperatures (Dikmen et al., 2008).

## Breed Diversity and the *SLICK* Gene

*Bos Indicus* (Indicine) cattle originated in the Indian sub-continent but have now spread throughout the World (Utsunomiya et al., 2019), resulting in several sub-species. These animals are relatively thermotolerant for various reasons (Turner and Schleger 1960; Hansen, 2004) including short hair, large skin folds, increased heat dissipation from the body to the skin and lowered metabolic rate, although reports of their possessing more sweat glands and having a greater sweating rate are controversial. The genetic basis of thermotolerance in *Bos Indicus* or breeds derived from the same has been a subject of considerable investigation (Hansen, 2004; Pires et al., 2021). In 2004, Hansen (Hansen, 2004) wrote “Almost nothing is known about speciﬁc genes controlling heat resistance in zebu cattle and, indeed, our understanding of the physiological basis for thermotolerance in these cattle is still incomplete.” Since then, however, considerable progress has been made (*vide infra*).

Extensive genetic differences are now evident between *Bos Taurus* and *Bos Indicus* (Bolormaa et al., 2013), the former being relatively less thermotolerant. A recent paper compared copy number variation (CNV) between *Bos Taurus* and *Bos Indicus* highlighting essential genome diversity between the two (Hu et al., 2020). For example, this study revealed a 1.74 Mbp indicine-specific sequence that could only be mapped to *Bos Indicus*. The differentially expressed genes are thought to be functionally involved in heat tolerance, metabolism, and other traits associated with adaption to warmer climates.

In addition to the widespread differences between *Bos Taurus* and *Bos Indicus*, it is apparent that cattle of composite breeds are thermotolerant and have short, shiny coats (Olson et al., 2003; Dikmen et al., 2008, 2014). Thus, a mutation of a dominant gene, mapping to chromosome 20 in Senepol-derived cattle, in a region of the chromosome that includes coding for the PRL receptor (Mariasegaram et al., 2007) was found to associate with the *SLICK* phenotype.

## Breeds Displaying the SLICK Phenotype

### Senepol

The Senepol breed originated in St Croix, a county of the US Virgin Islands in the early 1900s, through a breeding objective of Henry Nelthropp and family to develop a hornless, early maturing breed with a mild disposition and adaptability to tropical climes (Flori et al., 2012). The cross was between cows presumed to N’Dama originating from Senegal and local Red Poll bulls originating animals of Great Britain. The true genetic origin of the N’Dama breed has been questioned although the presence of the *SLICK* gene was verified in Senepol (Flori et al., 2012). The breed, sourced from various populations, was found to be 90% European ancestry and 10% Zebu, strongly suggesting that the original cross did not incorporate N’Dama to any significant extent. These authors also identified a region on Chromosome 20, that conferred thermo-tolerance.

A significant advance was the identification of a point mutation in the PRL receptor gene that was associated with the *SLICK* phenotype in Senepol cattle. Thus, based on the reasonable premise (Mariasegaram et al., 2007; Flori et al., 2012; Huson et al., 2014), Littlejohn et al (Littlejohn et al., 2014) interrogated the genetic makeup of Senepol cattle displaying the *SLICK* phenotype. They identified a single base deletion in Exon 10 of the PRL receptor gene that introduces a premature stop codon (p.Leu462*) and loss of 120 C-terminal amino acids from the long isoform of the receptor.

Examining other breeds (including Bos Indicus), it was apparent that the mutation was unique to Senepol, so the short-haired Indocine phenotype and its association with thermotolerance appears not to be due to the mutation identified in Senepol. As the authors pointed out, the link between the mutation and thermotolerance remained to be elucidated. More recently, as discussed below, other mutations in the PRL receptor, which do not occur in *SLICK* Senepol, have been reported in *SLICK*-coated Limonero cattle (Porto-Neto et al., 2018) (*vide infra*). An open question remains as to how the mutation(s) in the PRL receptor actually confers thermotolerance in Senepol cattle. Notably, however, this breed does not display any difference compared to wild-type animals in the number of sweat glands in the skin. Thus, Sosa et al. (2022) found no difference in the total area of sections of skin occupied by sweat glands between cows heterozygous for *SLICK* and those without the mutation. It was concluded that the *SLICK* mutation might affect the function of the sweat glands because of the greater level of immunoreactive *FOXA1* found in the sweat gland epithelium. Sweat glands express *FOXA1* which regulates the Bestrophin-2 gene (*BEST2*), the latter being necessary for the secretory function of the sweat glands (Cui et al., 2012). As indicated above, this may represent a gain-of-function that is yet to be defined. Additional experiments are warranted to determine whether the amount of FOXA1 in the sweat gland epithelium affects sweat gland function and whether, as speculated by Foitzik et al. (Foitzik et al., 2009), the sweat gland is one of the physiological targets of PRL in cattle, as in sheep (Choy et al., 1995, 1997). As to how sweating is regulated in *SLICK*, and indeed other animals/species, requires further elucidation.

It must be noted that, whereas the *SLICK* gene (PRL receptor mutation) has been the subject of considerable focus, many other genes are likely to be important in the development of thermotolerance as well as within Senopol; *SKP2* (S-phase kinase-associated protein 2) for example (Huson et al., 2014).

### Criollo/Limonero

Another breed of interest is the Venezuelan Criollo or Criollo Limonero, which is Bos Taurus. The breed is descended from cattle of southern Spain (Andalusia), taken to South America. Over 400 years, these cattle have adapted to the Venezuelan climate. The breed was threatened by extinction, but a herd was maintained at the Instituto Nacional de Investigaciones Agropecuarias, Zulia, Venezuela. The reason Criollo Limonero seems well-adapted to the tropical climate of Venezuela is thought to be related to their hair type and blood flow physiology. Selection has favored the slick hair type, with relatively greater heat dissipation from the skin. Criollo Limonero displays features such as loose skin, similar to zebu, which may confer thermotolerance (Landaeta-Hernández et al., 2011). Sweat glands in Slick-haired animals of this breed were greater in size than in normal-haired animals of the same breed and the shape of the glands was different; the Slick-haired had glands with a wider lumen, thick walls, and abundant cells whereas the normal-haired animals had long, thin sweat glands (ibid). As indicated above, the Limonero displays mutations other than that identified in Senepol, which raises the question as to whether Slick phenotype is due to one or more mutations, all within the PRL receptor gene (Porto-Neto et al., 2018).

### Carora

The Carora breed originates from a cross between Criollo and Brown Swiss cattle in Venezuela, in the 1930s with selection favoring the *SLICK* gene phenotype (Cerutti et al., 2006). Carora also possesses the *SLICK* mutation, derived from Criollo.

## Introgression of the PRL Gene

As pointed out by Hansen (Hansen, 2020), the transfer of genes such as *SLICK*, offers a good means of mitigating HS in animals of high productivity, without the disadvantages of cross-breeding, which can compromise the same. This is preferable to selecting animals with lower core body temperatures because of the correlation between body temperature and milk yield (Dikmen et al., 2012). Introgression of other genes that confer heat resilience may also confer heat resilience, but the current information on the transfer of *SLICK* reinforces the notion that PRL signaling plays a fundamental role in the consequences and mitigation of HS.

## Other Candidate Genes for Heat Resilience

It is clear that the genomes of Indocine and Taurine cattle are very different, although there is some degree of overlap (Naji et al., 2021). Given that thermotolerance is clearly greater in Indocine cattle, there is further opportunity to exploit this by further interrogation of the genome and identification of particular genes that confer heat resilience. The studies of Kim et al (Kim et al., 2016) examined genotypes of Barki sheep and goats in a hot arid region of the western desert in Egypt, as well as the genotypes of a number of breeds from temperate climes. Selection signatures relating to adaption to a hot dry environment were found, including those for traits relating to thermo-tolerance, body size and development, metabolism, and immune function. Thus, natural or artificial selection in such an environment may favor particular genes that warrant further attention.

Interestingly, there are genes common to sheep and cattle that have been identified as being associated with heat resilience, such as *PCLB1* (Kim et al., 2016; Taye et al., 2017; Freitas et al., 2021). Freitas et al (Freitas et al., 2021) examined 3 breeds of Chinese cattle and identified mutations in several genes that related to thermotolerance, based on haplotyping, including *SLC9A4* and *ITPR2* (involved in thermal sweating), *PLCB1* (which hydrolyzes phospholipids into fatty acids and other lipophilic molecules), and *FTO* (which regulates metabolism). Selection pressure for these genes may confer thermotolerance. Taye et al. (2017) examined the genomes of five breeds of African cattle and identified several genes, using haplotype diversity, that may relate to heat resilience and that may have been subjected to positive selection pressure. These included *SOD1*, *GPX7, GSTM2, GSTM4, SLC23A1*, *SLC23A1 PLCB1,* and *FTO.* Interestingly, Kim et al. (2016) also identified *PLCB1* as a gene under selection in Barki sheep and goats (*vide supra*), raising the possibility that selection for these genes may be used as a marker for thermotolerance, although further work is required to consolidate the notion. Nevertheless, the studies discussed above raise the possibility that a single blood sample and harvest of white blood cells could enable the screening of many animals to identify specific candidate genes.

## Other Mechanisms Whereby Prolactin may Function during HS

### Prolactin, leptin, and food intake

A universal response to HS is a reduction in food intake, seen in mice (Morera et al., 2012), cows (Baile and Forbes, 1974; Rhoads et al., 2013), sheep (Joy et al., 2020) pigs (Cervantes et al., 2016), and goats (Lu, 1989). Nevertheless, in thermotolerant animals, a reduction in food intake is not seen, since reduction under HS was evident in merino cross-bred sheep, subjected to cyclic HS for 2 wk (38 to 40 °C between 0800 h and 1700 h daily) but not in thermotolerant Dorper sheep (Joy et al., 2020). Neither was food intake reduced in Bos Indicus cattle when the ambient temperature was raised from 20 °C to 32 °C for 5 d, whereas a marked reduction was seen in Bos Taurus (Beatty et al., 2004).

As to how the effect of HS on food intake is due to PRL action is not clearly delineated, but there is good circumstantial evidence that the anorectic hormone leptin may play a role. For example, PRL (produced by pituitary grafts or administered PRL) stimulates leptin gene expression in white adipose tissue and increases circulating concentrations of leptin in female rats (Gualillo et al., 1999), so an effect of HS to increase leptin concentrations, through PRL action, would hardly be surprising. Interestingly, other studies conducted in vitro on adipose tissue from male and female rats showed that PRL inhibited lipolysis and reduced leptin secretion (in epididymal fat cells), in concert with upregulation of the long and short forms of the PRL receptor (Brandebourg et al., 2007). Strangely, these authors did not cite the earlier study of Gualillo et al (Gualillo et al., 1999). In mice, an increase in plasma leptin concentrations is observed under HS (Morera et al., 2012) although the same was not the case for pigs (Cervantes et al., 2016). Data from other domesticated animals is wanting. Plasma leptin concentrations increased slightly (albeit significantly) with HS in Salem Black goats but not in Osmanabadi goats, the former being relatively more thermotolerant (Archana et al., 2018), but any association with food intake was not reported. There are minimal data for ovine or bovine as to how HS may affect leptin concentrations associated with a reduction in food intake, but this would be a worthy area of investigation. The effect of HS on leptin and adiponectin concentrations is not clear for bovine; although the paper of Bernabucci et al (Bernabucci et al., 2010) is often cited, the study was of photoperiodic effects, so the influence of temperature/humidity is confounded. One recent publication showed no difference in the expression of the leptin gene in Caracu (Bos taurus) and Nelore (Bos indicus) bulls under HS (Pires et al., 2021). To the knowledge of the authors, there is no evidence that a reduction in food intake seen under HS is due to an increase in leptin concentrations. In this regard, it seems most likely that environmental effects on leptin concentrations are more important than breed differences in the same (Pires et al., 2021).

### Prolactin and thermogenesis

Thermogenesis is a process through which facultative or adaptive processes lead to the generation of heat, particularly in brown adipose tissue (BAT) but also in muscle and beige adipocytes (Fuller-Jackson et al., 2017). BAT is active in ruminants as in other species (Henry et al. 2017), especially prevalent in the brisket of sheep.

Thermogenesis is a form of energy expenditure that can be categorized as:-

- Basal Metabolic Rate (BMR)
- Physical Activity
- Non-exercise activity thermogenesis (NEAT)
- Adaptive Thermogenesis including diet-induced thermogenesis

**Figure 3.**
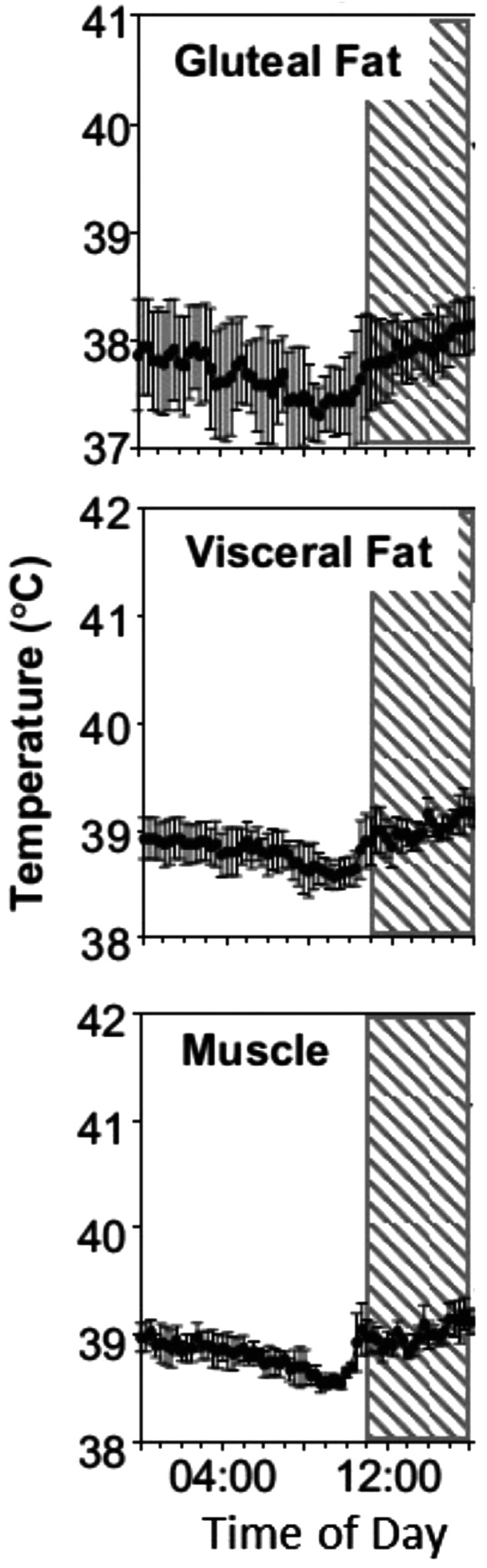
Tissue-specific post-prandial thermogenic profiles (means ± SEM) of gluteal fat, retroperitoneal fat, and skeletal muscle (*n* = 4/group). The hatched area indicates the time when food was offered. Reproduced from Henry et al (2008), with permission.

Brown adipocytes are rich in mitochondria and express uncoupling protein1 (UCP1), which “uncouples” the electron chain in the mitochondria, leading to the generation of heat. The process is activated in cold environments, to maintain body temperature. BAT is innervated by the sympathetic nervous system and thermogenesis is stimulated by noradrenalin, as well as adrenalin. BAT is also richly vascularized and thermogenesis is also stimulated by a range of hormones and substrates. It is clear from studies in sheep that thermogenesis is stimulated in muscle, as well as fat by feeding (diet-induced thermogenesis (Henry et al., 2008). Thermogenesis leads to an increase in core body temperature, so it is ostensibly relevant to the condition of HS. Whereas this could be due to a rise in leptin concentrations, which stimulates thermogenesis (Henry et al., 2008) (Figure 3), elevated circulating concentrations of adrenalin are also seen under HS in cattle (Alvarez and Johnson, 1973). Differences in plasma concentrations of adrenalin and noradrenalin have been reported for different sheep breeds (Horton and Burgher, 1992), but how these factors would influence thermogenesis under HS is unknown.

As to whether PRL is important in regulating thermogenesis, one study on sheep kept at 43 to 44 °C showed that treatment with CB154 (bromocriptine), an inhibitor of PRL secretion caused a significant rise in core body temperature (Salah et al., 1995), although PRL concentrations were not measured and whether there was an effect on BAT thermogenesis was not indicated. Other studies also indicate that PRL can inhibit thermogenesis (Yahata and Kuroshima, 1994). In particular, greater concentrations of PRL reduce concentrations of UCP1 in BAT during lactation in the rat (Chan and Swaminathan, 1990). Regarding the role of PRL in the regulation of thermogenesis in domestic animals, few studies have been done, but the rise in adrenalin that occurs under HS could be a stimulus.

### Cortisol, prolactin and heat stress

Cortisol concentrations are often raised during HS (e.g., (Sivakumar et al., 2010), leading some authors (Endris and Feki, 2021) to assert that “cortisol is the primary biochemical marker for HS in ruminant livestock,” but see below. This may depend upon ambient temperature and humidity, the length of the stressful period, and the susceptibility of individuals to the environmental stress. Short-term elevation of cortisol concentrations in species such as sheep may be beneficial in terms of an acute reaction but long-term elevation is considered detrimental (Hough et al., 2013). Importantly, HS (42 °C) of Malpura ewes increases cortisol concentrations, which is attenuated by antioxidant supplementation (Sejian et al., 2014) and the same is seen in goats subjected to HS (40 °C), in which increased concentrations of cortisol were lowered by antioxidant treatment (Sivakumar et al., 2010).

Cortisol concentrations are a sensitive indicator of HS in humans, but only when “discomfort” is indicated indicated some habituation is also observed following repeated daily HS (Follenius et al., 1982). On the other hand, HS has been shown to cause a chronic elevation in cortisol concentrations, as seen in sheep even at an ambient temperature of 30 °C, that is not diminished over a period of weeks (Wojtas et al., 2013), potentially leading to a range of harmful effects (*vide infra*). The degree of increase in cortisol concentrations seen in sheep under HS is strongly correlated (*r*^2^ = 0.48) to the temperature-humidity index (THI) (Slimen et al., 2019). Importantly, however, the increase in cortisol concentrations seen with HS (THI > 80) in dairy cows is slightly increased (albeit significant) in cows with greater sensitivity to the stress than in those with lesser sensitivity (based on rise in rectal temperature) (Chen et al., 2018). Despite there being many reports of a rise in plasma cortisol concentrations under HS, there are also several examples where there is either no change (Chauhan et al., 2014b) or a reduction in concentrations. Thus, cortisol concentrations were seen to fall in neonatal pigs when the ambient temperature was raised from 18 °C to 34 °C (Carroll et al., 2001). In an early study of non-lactating Holstein cows, ambient temperatures of 40 °C or 43 °C caused an acute rise in cortisol concentrations, which returned to near-normal within 3.5 h (Alvarez and Johnson, 1973). The same study also showed an acute rise and then a decline in plasma cortisol concentrations over 72 h at an ambient temperature of 35 °C. A more recent study of young Holstein heifers maintained in either thermal comfort (THI 65) or hotter temperature (THI 84) for 17 d (Ronchi et al., 2001), showing a decline in cortisol concentrations at the warmer temperature (Figure 1). Importantly, in the same study, PRL concentrations were seen to rise, when cortisol concentrations fell, suggesting that there is no positive correlation in the concentrations of the two hormones.

The relationship that cortisol concentrations have to concentrations of PRL is not addressed in most studies, but it would be interesting to obtain a greater knowledge of the degree to which the concentrations of the two hormones are related during HS. For example, a study in goats showed a concomitant rise in both hormones during HS (Sivakumar et al., 2010), but any interaction between the two was not elucidated. A precedent for such a likely association is that a positive association between PRL and cortisol concentrations was seen in PCOS women, but indicated that more research is required to substantiate a causative role of the former on the latter (Glintborg et al., 2014).

Another factor that may be causative of increased cortisol concentrations under HS is the reduction in food intake, since concentrations increase with food restriction or fasting (Henry and Clarke, 2007). As indicated above, food intake may be affected by HS in some circumstances but not others, perhaps associated with a rise in PRL concentrations although the link with cortisol concentrations is not fully explored.

### Metabolism, prolactin, and heat stress

A recent review indicates that PRL has a significant role in regulating metabolism (Pirchio et al., 2022). In particular, mild elevation in PRL concentrations has beneficial effects on metabolic function in humans, acting on the pancreas, liver, adipose tissue, and the hypothalamus (Macotela et al., 2022). In support of this concept, McCarty et al. (2009) found that thermal therapy had the potential to enhance insulin sensitivity in humans. Furthermore, HS has been found to improve biomarkers of insulin sensitivity in diabetic rats (Kokura et al., 2010) and in rats on a high-fat diet (Gupte et al., 2009). The association between increased glucose-induced insulin secretion and insulin sensitivity appears to be also the case in ruminants.

It is clearly apparent, that the rise in PRL concentrations that occurs with HS in domestic animals is associated with reduced food intake in most cases, although this is breed-dependent, as indicated above. In addition, several metabolic processes are impacted. PRL regulates the production of leptin and adiponectin (*vide supra*), that respectively inhibit and stimulate food intake, thus influencing metabolic balance. Despite the reduction in feed intake, plasma insulin and glucose concentrations are generally unchanged or increased (Joy et al., 2020; Prathap et al., 2022; DiGiacomo et al., 2023; Hung et al., 2023). Where plasma glucose has been reduced the decrease has either been quite small (Achmadi et al., 1993) or there has been either a large reduction in feed intake and/or an increase in plasma insulin (Wheelock et al., 2010; Hall et al., 2016). Also, a reduction in blood glucose is more likely to occur in lactating ruminants where there is a large glucose demand for synthesis of milk lactose (Wheelock et al., 2010; Hall et al., 2016). Therefore, it appears that HS influences glucose metabolism by increased insulin secretion and/or increased sensitivity in ruminants and pigs (reviewed in (Slimen et al., 2019)).

There is growing evidence that HS in ruminants and pigs increases insulin sensitivity. For example, Hung et al. (2023) found that warm/hot ambient temperatures increased basal plasma measures of insulin sensitivity. Also, the plasma insulin response to the intravenous glucose tolerance test (IVGTT) was decreased in sheep exposed to HS which is again indicative of improved insulin sensitivity in sheep. In support, Henry (2012) found that HS reduced insulin resistance in sheep. Similarly, Sanz Fernandez et al. (2015) found that insulin sensitivity was increased during HS in pigs. These observations are consistent with other findings in pigs where HS reduced the acute insulin-releasing rate and hence slowed the glucose clearance rate after an IVGTT (Liu et al., 2017). Although DiGiacomo et al. (2023) found that insulin resistance was increased in sheep during HS, the consensus is that insulin sensitivity increases during HS. Thus, enhanced insulin sensitivity may serve as a crucial aspect of the acclimation process during HS.

The augmentation of insulin sensitivity could correlate with reduced metabolic heat production, suggesting that preservation of insulin sensitivity is pivotal to thermoregulation, particularly under HS conditions. Therefore, maintaining glucose homeostasis presents a viable strategy to enhance adaptation to HS. Increased insulin sensitivity would ensure that heat-stressed animals would reduce adipose tissue lipid mobilization and diverting from the use of non-esterified fatty acids as a preferred energy substrate, despite the reduction in feed intake that generally occurs during HS. Rather, glucose would become the preferred energy substrate for peripheral tissues. HS causes a reduction in blood pCO2 and an increase in blood pO2, with a resultant decrease in base excess and an increase in blood pH in sheep (Gonzalez-Rivas et al., 2017; Joy et al., 2020). These changes in blood gas parameters are most likely a result of increased respiration rate and CO_2_ exhalation during HS. The use of lipids as a metabolic substrate during HS would result in a low respiratory quotient (Blaxter, 1962) and, therefore, further exacerbate the reduction in the blood base excess resulting in respiratory alkalosis. An increase in insulin sensitivity would inhibit lipolysis and fat mobilization whilst still ensuring hepatic gluconeogenesis since the effective dose to reduce plasma NEFA concentrations is within the physiological range and less than that which inhibits gluconeogenesis (Petterson et al. 1993, 1994).

In ruminants, however, rumination produces volatile fatty acids (VFA) which are taken up into the bloodstream to provide substrate for gluconeogenesis and PRL action in the liver, which is substantial (Kirsch et al., 2022). However, as pointed out by Ben-Jonathon et al. (Ben-Jonathan et al., 2006), surprisingly little is known about how PRL acts in the liver, but since it acts to regulate enzymes and transporters in other tissues, such as breast and adipose, one would expect the same in the liver. HS, however, mobilizes several reserves, which act as substrates for gluconeogenesis and could counteract the increase in insulin and insulin action. In particular, HS in ruminants causes increased blood concentrations of VFA, indicating increased protein and lipid catabolism, to meet increased energy demands (Salama et al., 2014; Slimen et al., 2016). There are also associated changes in hepatic glucose output through altered glycolysis, glycogenolysis, and gluconeogenesis, which is well discussed by Baumgard and Rhoads (Baumgard and Rhoads, 2013). A particular observation of note is that the expression of the pyruvate carboxylase gene is increased due to HS in the livers of lactating dairy cows, which would enhance gluconeogenesis (Rhoads et al., 2011). The extent to which this is totally or partly due to PRL action on the liver is unknown but may be related indirectly to changes in insulin sensitivity. The role of PRL in the liver is thought to be largely protective (Kirsch et al., 2022) and overexpression of the PRL receptor improves insulin sensitivity, whereas knockout of the receptor does the reverse, either in hepatocytes in vitro or in vivo (Yu et al., 2013). Many genes in the liver are either up- or down-regulated by the SLICK mutation in the PRL receptor, but none of these appear to relate to the gluconeogenic pathway (Sosa et al., 2022). Accordingly, increased PRL concentrations during HS could impact metabolism by direct action on the liver, but further work is required to demonstrate this.

**Figure 4.**
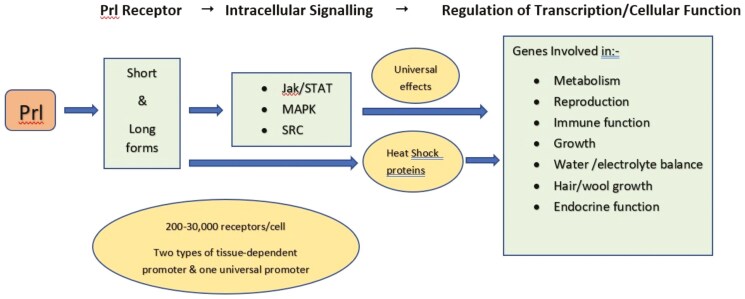
The effects of HS and the elevation of PRL concentrations may be direct or indirect, either by action on specific physiological processes. Through activation of the cognate receptor, PRL may act on multiple tissues and multiple physiological processes. This may be via activation of HSP.

The PRL receptor activates the JAK/STAT pathway of intracellular signaling, so any cell expressing the receptor and in which gene promoters possess the relevant cognate STAT binding site may be regulated by PRL (Bole-Feysot et al., 1998) clearly having implications in terms of control of metabolism. HSP70 induction by reactive oxygen species (ROS) occurs through activation of the promoter by enhanced binding of STAT5 to the specific binding site on the promoter. PRL induction of JAK/STAT signaling may do the same, conferring protective properties to the cell, limiting damage by ROS. Indeed, there is evidence for this in a PRL-dependent cell line (Blake et al., 1995). There is a close working relationship between heat shock factors (HSF) and HSP, which is delineated in a recent paper (Zhang et al., 2022) but it has not been studied in heat-stressed domesticated animals. In cattle, significant attention is paid to HSP70, which is the most abundant HSP (Beckham et al., 2004), which has been reviewed in relation to HS in cattle by Hansen (Hansen, 2020). Prospects for gene introgression or gene editing as a strategy for reduction of the impact of HS on production and reproduction in cattle have been discussed (Hansen, 2020). A summary of how HSP may affect changes in function during HS is shown in Figure 4. The involvement of PRL in the HS response may be direct or via activation of HSP, with downstream consequence. There are two variants of HSP70 in cattle, being HSPA1A and HSPA1L and a mutation in the 5’ untranslated region of the latter confers heat tolerance in bovine lymphocytes (Basiricò et al., 2011). Selection for such mutations may be a useful approach for the selection of heat tolerance in domestic animals.

The thyroid hormones are key regulators of metabolism (Mullur et al., 2014) and ambient temperature plays a key role in thyroid hormone function (Saber et al., 2009). Thyroid hormone concentrations are reduced by HS (Bernabucci et al., 2010). Although PRL and thyroid function are intimately linked, there is no available information as to how PRL might act on the thyroid to regulate function. Alternatively, thyroid function may affect PRL production, so altered levels during HS may be due, in part, to elevated concentrations of PRL under such conditions.

## Immune System and Heat Stress

It is well established that PRL acts on a number of cell types in the immune system (Bole-Feysot et al., 1998; Yu-Lee, 2002; Borba et al., 2018). In particular, it was stated some time ago that “PRL can be viewed as an immunopermissive trophic neuropeptide whose specific target is the T-lymphocyte” (Chikanza and Panayi, 1991). Thus, PRL promotes the survival and function of T lymphocytes (Krishnan et al., 2003). The action of PRL on the immune system is, however, complicated since it has been observed to have pro- and anti-inflammatory action in rheumatoid arthritis (García-Rodrigo et al., 2023).

Furthermore, HS, in concert with elevated PRL concentrations, perturbs the immune system as seen in a study by do Amaral et al. (2010). Thus heat-stressed cows displayed increased PRL concentrations and lymphocytes from heat-stressed animals showed lower proliferation, lower expression of genes for *PRL-R,* and greater expression of *SOCS-1* and *SOCS-3*. Expression of tumor necrosis factor-α (*TNF-α*) was lower in heat-stressed cows, perhaps due to increased *SOCS* expression. The authors concluded that cooling of the cows improved immune function and that the effects of HS might be attributable to PRL function. Min et al. (2016) reported increased TNFα and IL6 production with HS in dairy cows, indicating activation of the inflammatory cytokines.

Another study showed that circulating concentrations of IL-1ß, IL-6, IFN-γ, and TNF-α were increased in dairy cows with an increase in THI (Chen et al., 2018), emphasizing that assessment of the HS effect necessitates consideration of more than one cell type; these circulating cytokines could have origin in multiple cell types. In sheep, a specific effect is seen in the expression of pro-inflammatory cytokines (*TNFα* and *NFκB*) which was markedly increased in skeletal muscle when exposed to extreme HS, an effect that was ameliorated by administration of selenium (Chauhan et al., 2014a).

Greater response of the immune system during HS could lower the susceptibility to disease and one recent study (Cartwright et al., 2021) examined the function of blood mononuclear cells from dairy cattle classified as “high immune responders,” based on estimated breeding values. Cells from high immune responders had greater HSP70 values during HS than cells from average or “low immune responders” and displayed greater cell proliferation in vitro. A number of genes associated with HS and immunity have been identified in various tissues in GWAS studies (Lemal et al., 2023). Kim et al (Kim et al., 2016) studied sheep and goats in a hot arid environment and a number of exotic breeds not in the same environment, identifying *IL2, IL7, IL21,* and *IL1R1* as candidate genes for selection. High immune responders could be selected to be more tolerant to HS but the role of PRL in the determination of breeding value would be interesting to explore in depth. Whilst the role of PRL in the immune response to HS may be important, it is also salient to acknowledge the role that cortisol plays in the regulation of the immune system (Lemal et al., 2023).

## Endophytes, Heat Stress, and Prolactin

Endophytes in pasture plants, such as tall fescue and perennial ryegrass, produce ergot alkaloids, which can act as dopamine agonists (Aldrich et al., 1993; Aslanoglou et al., 2021). It is well established that consumption of ergot alkaloids from tall fescue and perennial ryegrass reduce circulating PRL concentrations while increasing rectal temperature and other signs of HS in sheep and cattle (Kerr et al., 1990; Porter and Thompson, 1992; Rice et al., 1997; Henry et al., 2016, 2019; Valente et al., 2024). Indeed, one particular study (Kerr et al., 1990) found a strong negative correlation between plasma PRL and rectal temperature in cattle consuming endophyte-infected tall fescue hay that disappeared under basal conditions when cattle were not consuming endophytes. Also, the effect of ergot alkaloids on plasma PRL concentrations and physiological responses such as rectal temperature and respiration rate are dose-dependent. Henry et al., (2016, 2019) found that plasma PRL concentrations were reduced by ergot alkaloids and increased by HS. Interestingly, the reduction in PRL concentrations associated with ergot alkaloid consumption was partially reversed during HS. Although no cause and effect can be established, this indicates that the effects of HS on plasma PRL are still evident in animals where dopamine agonists inhibit plasma PRL concentrations. Valente et al. (2024) recently found that feeding a dopamine precursor (levodopa, L-DOPA) could partially reverse the effects of ergot alkaloid consumption on plasma PRL concentrations in mildly heat-stressed steers. Interestingly, the L-DOPA-mediated increase in PRL in steers was associated with an increase in glucose clearance, lending support to the role of PRL in improving insulin sensitivity during HS, as indicated earlier. However, earlier research (Aldrich et al., 1993) did not observe a reversal in the reduction in plasma PRL concentrations in response to the consumption of ergot alkaloids in lambs fed a dopamine agonist even though feed intake was ameliorated.

## Water and Electrolyte Balance

Numerous studies indicate an effect of HS on water and electrolyte balance which will not be recapitulated herein (Alamer, 2011; Taye et al., 2017). It is clear from these studies that this may involve PRL signaling, but more needs to be done to firmly establish the link between increased PRL concentrations during HS and water and electrolyte balance.

## Conclusion

**Figure 5.**
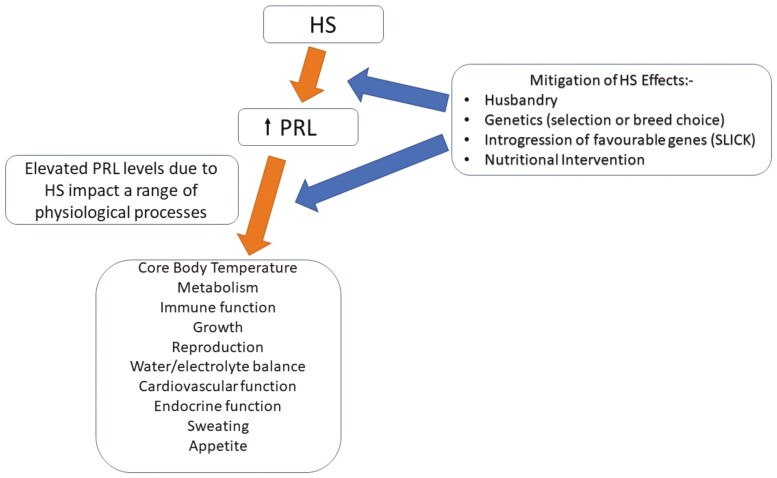
HS increases circulating PRL concentrations which impact on a range of physiological processes. Effects of HS may be counteracted in various ways which may or may not be via PRL action. A range of means of mitigation of the effects of HS are shown.

As indicated above, PRL performs multiple functions in the body, affecting a range of physiological processes. It may also be accepted that elevation of PRL concentration is a signature of HS (Figure 5). The identification of the *SLICK* gene and its ability to mitigate the effects of HS has been a significant and important advance in our understanding of the mechanisms that promulgate the HS response, but the exact means by which this is effected remains to be elucidated. Introgression of the *SLICK* gene is clearly an exciting and feasible means of counteracting HS (Davis et al., 2017), emphasizing the key role of PRL signaling in this stressor. Nevertheless, it may be fruitful to identify genetic variants within or between populations that demonstrate greater and lesser PRL responses to HS and ascertain how this correlates with the degree of stress. For example, Dorper (blackface) sheep display a greater rise in PRL concentrations on hot days (compared to cool days) than whiteface Dorpers (Joy et al., 2022b), a preliminary finding that requires substantiation and investigation as to how such a difference may confer heat resilience in the Dorper. With respect to differences between breeds, Dorper sheep display a greater increase in liver expression of interleukin2 and *PRLR* than do cross-bred merino sheep in response to HS, providing an example of difference between breeds that needs further investigation to ascertain how PRL plays a role (Joy et al., 2022a).

Another area of fruitful investigation is the use of nutritional supplements to mitigate HS. Chromium and betaine have proved useful in pigs (Cottrell et al., 2015) and selenium and vitamin E are efficacious in sheep (Chauhan et al., 2014a, 2015). Betaine and chromium have beneficial effects on cattle (DiGiacomo et al., 2014). As to how PRL action might be involved in the processes that lead to the alleviation of HS by such nutritional interventions could be informative.

Finally, it should be kept in mind that increased concentrations of PRL induced by stress in humans may be diminished by habituation, which could occur in cases where HS is prolonged (Fava and Guaraldi, 1987). Horowitz (Horowitz, 2017) reviewed this particular issue, highlighting the role of epigenetics in Heat Acclimation-Mediated Cross-Tolerance, which explains how humans who move into hot climates may acclimate. As to how PRL might be involved in habituation has not been investigated.

## Abbreviations

BAT: brown adipose tissue
BEST2: bestrophin-2
BMR: basal metabolic rate
CB154: bromocriptine
FOXA1: forkhead box protein A1
FTO: fat mass and obesity-associated gene
GPX7: glutathione peroxidase 7
GSTM2: glutathione S-transferase Mu 2
GSTM4: glutathione S-transferase Mu 4
HPD: hypothalamo-pituitary disconnection
HS: heat stress
HSP: heat shock proteins
IFN-γ: interferon-γ
IL: interleukin
ITPR2: inositol 1,4,5-Trisphosphate Receptor Type 2
IVGTT: intravenous glucose tolerance test
JAK: Janus kinase
L-DOPA: levodopa
MAPK: mitogen-activated protein kinase
ME: median eminence
NEAT: non-exercise activity thermogenesis
NF-κB: nuclear factor-κB
PCLB1: phospholipase C Beta 1
PIT-1: pituitary-specific transcription factor
PRL: prolactin
ROS: reactive oxygen species
SKP2: S-phase kinase-associated protein 2
SLC9A4: solute carrier family 9 member A4
SLC23A1: solute carrier family 23 member 1
SNP: single-nucleotide polymorphism
SOC: suppressor of cytokine signaling
SOD7: superoxide dismutase type 1
STAT: signal transducer and activator of transcription
THI: temperature-humidity index
TNF-α: tumor necrosis factor-α
TRH: thyrotropin-releasing hormone
UCP1: uncoupling protein1
